# Azoturia

**Published:** 1897-06

**Authors:** John R. Hart

**Affiliations:** Philadelphia


### AZOTURIA.

By John R. Hart, V.M.D.,
PHILADELPHIA.

(Continued from page 286.)

*Urea.* As to why and how urea gets into the blood. It is known that the albuminous food constituents in digestion, through a process of hydration by the action of ferments, are converted into peptones which enter by absorption into the radicals of the portal vein, but an insignificant portion being absorbed by means of the lacteals when once within the blood current, possibly in the process of absorption itself; the peptone must apparently be reconverted to the form of albumin, for the amount of peptone detected in the portal vein, even after an abundant albuminous diet, is too small to represent the amount of albuminous matter absorbed. We may, therefore, assert without fear of contradiction, that almost immediately upon entering the blood current the albuminous food constituents are converted mainly into serum albumen. It has further been stated that urea represents the final product in the series of decompositions which the albuminous bodies of the tissues undergo. The attempt to trace the changes, commencing with albumin and terminating in the production of urea, is frustrated by the mysteries of the process. It is known that with the withdrawal of all food the excretion of urea decreases. It is further clear that urea is formed at the expense of the albuminous constituents of the tissues; that the excretion of urea may be increased by albuminous food, and the amount of urea eliminated increases proportionately with the increase in albumin administered as food. Urea is not, however, a simple product of oxidation of albuminous bodies, but is a product of complicated decompositions.

An increase in muscular exercise leads to an increase in the elimination of urea. As the amount of kreatin formed by the muscles increases with exercise, and the amount of urea eliminated by the kidneys increases under the same circumstances, it would appear reasonable to assume that the kreatin resulting from the breaking down of the albuminous tissue constituents of muscles and nerves represents the main source of urea. The suppression of urine is followed by the accumulation in the system of a large amount of urea. Again, the urea under all circumstances in this disease becomes a constituent of the blood. Another source of urea may be found in the products of decomposition of albuminous matter in the intestine. The introduction of a large amount of proteid in the alimentary canal is followed by a corresponding increase in the amount of urea eliminated.

An excess of proteid over and above that needed for nutrition in the alimentary canal breaks down under the influence of pancreatic digestion into leucin and tyrosin.

If leucin be itself introduced into the intestinal tubes the amount of urea eliminated will be proportionately increased. Leucin, therefore, may represent a step in the process of conversion of albumin into urea. In the latter case we can probably locate the conversion of leucin into urea in the liver, for the liver, unlike other glands, normally contains large quantities of urea.

We can assume, perhaps, that leucin is absorbed by the portal vein, hence the formation of urea out of leucin would be a natural conclusion.

This evidently shows the relation between the liver and the kidney in the disease of azoturia.

*Nerves.* As to the nerves. Complex causes which are capable of introducing alteration fall often upon the nerve elements, and through them, as an intermediary, provoke reflex disturbances. Some manifest themselves in the diseased part or organs, where the irritation of blood absorption and nutrition causes work to be done in an abnormal manner. Others reflect upon the whole economy; the heart and respiratory action is accelerated, and the elaboration of material is found altered in all the cells of the body. Urea and carbonic acid are produced in much greater quantity. Nerve reaction has not only added certain peculiar features to local manifestations, it has bound the whole organism to the work which is going on in the diseased part. Besides, nerve reaction nearly always borrow the coöperation of another. Pathogenic process, every nerve excitation, cold, shock, emotional or traumatic, may produce alterations of some kind. These are not diseases. The real disease, when it is roused by nerve reaction, infers, except in cases where the stimulation is excessive, and those wherein the nervous system is abnormally excited, an essential deterioratiou of the organism. A disturbed nervous system induces disturbance of nutrition, perverted nutrition leads to the development of new substances which may become toxic. There are often formed in the organism peptones which do not originate in the intestinal tube, but which are injurious in this sense, that escape by the urine, and thus bring about an abnormal spoliation of the organism. There are thus produced abnormal albumins which escape by the kidneys and seem capable of destroying the nutrition of the renal epithelium and of introducing certain nephritis.

Disease also causes the appearance of abnormal coloring matter or of substance transformable into coloring matter, among which are found those that in urine take on a red coloration under the influence of perchloride of iron, as leucin, tyrosin, and all the imperfect excrementitious products which arise from insufficient elaboration on the part of the liver, and many other toxic substances.

*Bladder.* The bladder also plays an important part in this disease, as distension of the bladder starts sensory impulses which are conducted to the spinal cord through the posterior roots of the third, fourth, and fifth sacral nerves.

As soon as the bladder becomes distended the sphincter becomes mechanically dilated, and the retained urine is apt to undergo ammoniacal fermentation. This evil you can remedy somewhat by the use of the catheter as often as you think necessary.

*Urine.* Now, as to what may be discovered from the urine in conjunction with this disease.

According to chemistry, the great number of high or low colored urines or any alteration in urine are not original products, but result from decomposition of urea and uric acid, and the recomposition of these into new forms. The slightest disturbing influences seem capable of producing these changes. There is a wide difference between the physical character of sugar and that of oxalic acid, and between that of albumin and of uric acid, and yet they have so close a resemblance in their chemical composition as is generally sufficient in a single element. One of them should be sufficient to create another. Such attempts appear still more likely to be unsuccessful when it is considered that the morbid state of the urine is for the most part an effect rather than a cause of disease—the result either of inflammation or some structural lesion of the kidney, or some constitutional trouble.

Still oftener the case is some derangement of the digestive function, the primary assimilation of the food may be imperfect, and hence the whole function of nutrition becomes distorted.

Modes of detecting in ordinary practice the most important alteration of urine may not be misplaced here. A simple method consists of a gravimeter for measuring the specific gravity of fluids, a little blue and red litmus paper, a test tube, a spirit-lamp, and a vial of nitric acid.

The urine, if acid, will redden the blue litmus paper; if alkaline, it will turn the red paper blue. If neutral it will produce no change in either color. If there is no cloud in the urine a small quantity may be placed in the test tube over a spirit-lamp. Should a white deposit be formed it must be either albumin or the earthy phosphates. The addition of a drop of nitric acid will coagulate the albumin more firmly, but dissolve the salts. If the urine is very high colored, it may be supposed to contain either coloring.matter or bile or blood or an excess of uric acid. If blood be present, heat will cause the liquid to lose its transparency, as will also nitric acid; whereas heat will not effect the bile-pigment, and nitric acid will at once turn it green. If uric acid be the cause of the dark color of the liquid examined, the addition of nitric acid will precipitate it in the form of a brownish sediment. Urine made green by the addition of nitric acid contains coloring-matter of bile. Mucus in urine, if much, is generally owing to vesical catarrh or to enlargement of the prostate gland. Pus may be derived from inflammation of the pelvis, of the kidney, or from the lining membrane of any part of the urinary passages, or from abscess of kidney or prostate gland.

*Muscles.* The characteristics of muscles should be taken into consideration in this disease. In the first place, in a state of rest living muscle has an alkaline reaction; in dead muscle and in muscle in contraction, the reaction is acid, from potass, phosphate, and carbonic acid. Living muscle is to a certain extent translucent, extensible, and elastic; dead muscle is opaque, rigid, inextensible, and has lost its elasticity. This is due to coagulation of myosin. Why some animals remain lame and do not recover their normal condition of muscles in the posterior parts is due to a condition of fatigue, due either to insufficient supply of nutritive substance or to the accumulation of the products of decomposition, which produce a harmful aetion on the muscle-fibres. When a muscle contracts its arterioles dilate, more blood passes through the muscle, and as a consequence the removal of the increased carbon dioxide formed is facilitated. For instance, in this loss of power to these muscles, when contracted thus, muscles arising in one bone and inserted in another, will in their contraction either move both bones toward each other, or if one be fixed will draw the movable to the fixed bone. I think in these cases of azoturia the psoas muscles and abductors are as much in fault as the crural muscles, as they have their insertion almost perpendicular to the bones on which they act. As you know, in the posterior part of the horse four angles are met with: the hip-joint, the patella, hock, and pastern-joint. The pelvis is very oblique relatively to the trunk of the horse, the femur is obliquely connected with the pelvis, downward motion of the pelvis, a backward motion of the femur from the body weight being prevented by the abdominal recti muscles, whose tendons, inserted in the pelvis, by their tension, tend to draw the hip-joint anteriorly. The gluteal muscles arising from the ilium and passing over the hip-joint to the femur, act in the same direction, not only preventing forcing backward of the hip-joint, but in its contraction pressing the hip-joint forward, so the leg is like the femur, flexed, its obliquity being limited by the tension of the tendons of the extensor muscles which pass over the anterior surface of the limb.

Often a rupture of fibres in the psoas muscles will prevent an animal from getting up on his feet, and frequently in this disease these muscle-fibres are ruptured.

*Skin.* As to the skin and what assistance it gives in this disease in assisting nature to throw off the poisonous material.

The skin may be regarded as an organ supplementary in its action to the lungs and kidneys, since the skin by its secretion is capable of removing a considerable quantity of water from the blood, small amounts of carbon dioxide, and small amounts of salts, and in certain instances, during suppression of renal secretion, a small amount of urea.

The skin is thus an excretory organ serving to remove gaseous, liquid, and solid waste products.

It is also influenced by the mental condition, by medicines, by poisons, and under the head of renal secretion the activity of the skin as a secreting organ is, as a rule, supplementary to that of the kidney.

(To be continued.)

